# *Leaf Mutant 7* Encoding Heat Shock Protein *OsHSP40* Regulates Leaf Size in Rice

**DOI:** 10.3390/ijms23084446

**Published:** 2022-04-18

**Authors:** Fuhua Wang, Zhengbin Tang, Ya Wang, Jing Fu, Wenbo Yang, Shengxuan Wang, Yuetao Wang, Tao Bai, Zhibo Huang, Haiqing Yin, Zhoufei Wang

**Affiliations:** 1 Institute of Cereal Crop, Henan Academy of Agricultural Sciences, Zhengzhou 450002, China; wangfuhuahunan@163.com (F.W.); wangya840212@163.com (Y.W.); fujing8210@sina.com (J.F.); bbg.123@163.com (W.Y.); 13017671205@163.com (S.W.); wangyuetao2012@163.com (Y.W.); baitao197119@163.com (T.B.); 2 The Laboratory of Seed Science and Technology, Guangdong Key Laboratory of Plant Molecular Breeding, South China Agricultural University, Guangzhou 510642, China; rudytang@aliyun.com (Z.T.); 20202015013@stu.scau.edu.cn (Z.H.)

**Keywords:** rice, leaf size, heat shock protein, natural variation

## Abstract

Leaf size is an important agronomic trait directly affecting yield in rice, and thus understanding the genes determining leaf size is important in breeding. In this study, one *Leaf Mutant 7* (*lm7*) with small leaf size was isolated using ethyl methane sulphonate (EMS) mutagenesis from the *japonica* Zhenggeng 1925. MutMap by whole genome resequencing of phenotypic bulks revealed that *LM7* is likely located in the 133 kb region on chromosome 7 using F_2_ population from a cross between *lm7* and wild-type (WT) Zhenggeng 1925. The candidate gene encoding heat shock protein *OsHSP40* for *LM7* was functionally validated. Disruption of this gene in *Oshsp40* mutants significantly reduced the leaf size compared with that of WT in rice. Microscopic examination showed that *OsHSP40* modulated leaf size via regulating the veins formation and cell size/cell number. Nucleotide diversity analysis indicated that a single nucleotide polymorphism (SNP) variation of C to T in the coding region of *OsHSP40* may cause small leaves among rice accessions. Therefore, the natural variation of *OsHSP40* contributing to leaf size might be useful for rice breeding.

## 1. Introduction

Rice (*Oryza sativa* L.) is one of the world’s most important crops. With increasing population, the enhancement of grain yield is the main target in rice breeding [1]. The leaf is the primary organ of photosynthesis, and its morphological traits, such as size and shape, directly affects yield [2,3]. Therefore, optimal leaf size, including length, width and area, is an important objective of rice breeding [4]. The molecular mechanism determining leaf size is not well understood because it is a complex trait that is influenced by multiple genes and environments.

Several genes associated with leaf size have been cloned with mutants in rice. For example, a mutation in *NARROW LEAF 1*, encoding a plant-specific protein involved in polar auxin transport, results in narrow leaves with fewer longitudinal veins [5]. *NARROW LEAF 2* and *NARROW LEAF 3* encode an identical *OsWOX3A* transcriptional activator, and the mutant of *nal2*/*nal3* causes narrow leaves with fewer longitudinal veins and larger bulliform cells [6,7]. *NARROW LEAF 7* encodes an auxin biosynthesis YUCCA enzyme, suggesting an involvement of auxin in the regulation of leaf size [8]. *NARROW LEAF 9* encodes a protein homologous to the *Arabidopsis* ClpP6 subunit, and its mutant reduces the number of small vascular bundles in the leaf blades [4]. Several genes associated with leaf width involving in auxin pathway, such as *TRYPOTOHAN DEFICIENT DWARF 1 (TDD1)* [9], *OsGH3.5*, *OsARF19* [10], *OsSAUR45* [11], and *OsCHR4* [12], have been cloned in rice. Additionally, several expansin genes such as *OsEXPA8* and *OsEXPB2* affect leaf width via cell expansion and have been isolated in rice [13,14]. Altogether, leaf size is controlled by the complex coordination of cell division and expansion, and a reduction in the number of leaf veins is an obvious characteristic of leaf mutants in rice [15].

Heat shock proteins (HSPs), including HSP100 (Clp), HSP90, HSP70 (DnaK), HSP60, HSP40 (DnaJ), and small HSPs (sHSPs), have been widely reported as playing essential roles in both plant growth and abiotic stress tolerance [16,17]. In fact, HSP40 proteins are functional partners for HSP70s involved in various essential cellular processes, including protein folding/unfolding, assembly/disassembly, and degradation [18]. The overexpression of *HSP70* genes positively correlates with the acquisition of thermotolerance, and results in enhanced tolerance to salt, water and high-temperature stress in plants [19]. During the past few years, the investigation of *HSP40* on regulating plant growth and abiotic stress tolerance has gradually increased. For example, a novel mitochondrial HSP40 family protein BIL2 promotes plant growth and resistance against environmental stress in brassinosteroid signaling in *Arabidopsis* [20]. The soybean HSP40 homologue *GmDNJ1* improves normal growth and heat stress tolerance [21]. In rice, *OsHSP40* has been recently reported to regulate programmed cell death (PCD) of suspension cells under high temperature, and it has multiple functions in normal growth and abiotic stress tolerance [22]. However, the regulation roles of *HSP40* on leaf size remain unclear in rice.

In this study, one leaf mutant *lm7* was generated by ethyl methane sulphonate (EMS) mutagenesis from the *japonica* Zhenggeng 1925, which possesses small leaf size with short length and narrow width. MutMap approach revealed that *LM7* encodes the heat shock protein *OsHSP40*, and it was confirmed by *Oshsp40* mutants with narrow leaves. We observed that *OsHSP40* modulated leaf size through regulating the veins formation and cell size/cell number. The natural variation of *OsHSP40* contributing to leaf size might be useful for future rice breeding. 

## 2. Results

### 2.1. Phenotype Characterization of lm7

**Figure 1:**
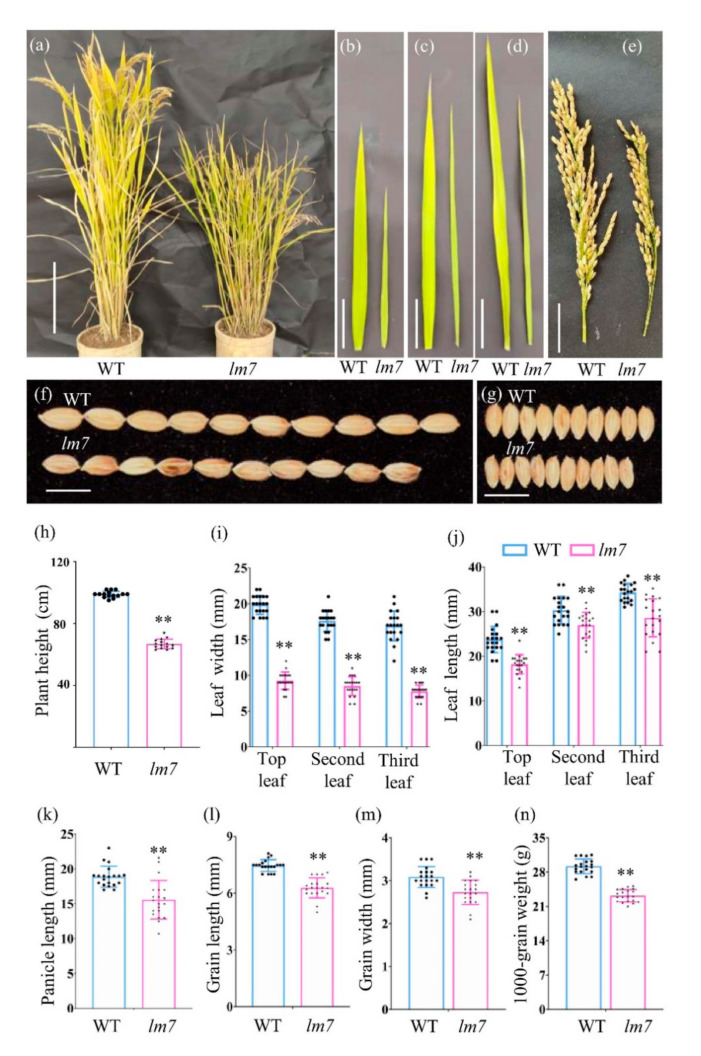
Phenotype characterization of *lm7*. (**a**) Representative image of mature plant of wild-type (WT) Zhenggeng 1925 and *lm7*. Scale bar is 20 cm. Representative image of the (**b**) top, (**c**) second, and (**d**) third leaf in WT and *lm7*. Scale bar is 5 cm. Representative image of the (**e**) panicle, (**f**) grain length, and (**g**) grain width. Scale bar is 1 cm. The mean value of (**h**) plant height, (**i**) leaf width, (**j**) leaf length, (**k**) panicle length, (**l**) grain length, (**m**) grain width, and (**n**) 1000-grain weight. Significant differences compared with the WT were determined using Student’s *t*-test: ** *p* < 0.01.

A leaf mutant 7 (*lm7*) was isolated from a population of *O. sativa japonica* Zhenggeng 1925 and mutated using a 0.9% ethyl methane sulfonate (EMS) solution. The phenotype of *lm7* was stably inherited after three generations of self-crossing. The *lm7* had shorter plant height and smaller leaves than wild-type (WT) Zhenggeng 1925 (Figure 1a–d). At the heading stage, the plant height of *lm7* was significantly lower than that of WT, which was decreased over approximately 35% plant height in *lm7* (Figure 1h). The width of the top, second, and third leaf in *lm7* was decreased by over approximately 50% compared with that of WT (Figure 1i), and the leaf length in *lm7* decreased over approximately 15% (Figure 1j).

To determine whether the *LM7* modulated yield traits, we investigated the panicle and grain traits of *lm7* plants. We observed that the panicle and grain traits in *lm7* were also different from those of wild-type (WT) Zhenggeng 1925. At the maturity stage, the primary panicle length of *lm7* was significantly lower than that of WT (Figure 1e). The panicle length of the *lm7* was 15.0 cm, while the panicle length of WT was 19.0 cm (Figure 1k). Both length and width of the grain in *lm7* were significantly lower than those of WT (Figure 1f,g). The grain length and width of *lm7* was 6.5 mm and 3.2 mm, respectively, while its corresponding WT was 7.0 mm and 3.6 mm (Figure 1l,m). The 1000-grain weight decreased significantly in *lm7* due to a change in the grain size. The 1000-grain weight of *lm7* was 24.0 g, while that of WT was 30.0 g (Figure 1n). Together, these results indicate that the *LM7* gene is likely a pleiotropic gene and plays an important role in yield.

### 2.2. Leaf Tissue and Cytological Observation of lm7

**Figure 2:**
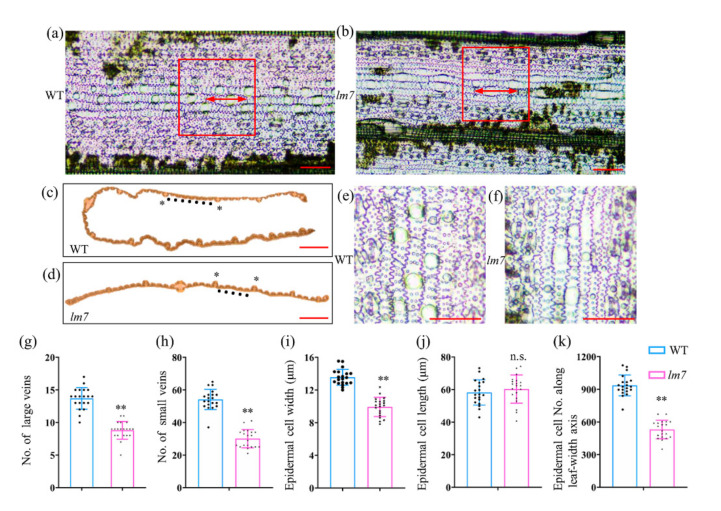
Leaf tissue and cytological observation of *lm7.* Representative image of epidermal cells of (**a**) wild-type (WT) Zhenggeng 1925 and (**b**) *lm7* leaves. Scale bar is 50 μm. Cross section of (**c**) WT and (**d**) *lm7* leaves. Asterisks represent large veins and solid circles represent small veins. Scale bar is 1 mm. A clear show of epidermal cells of (**e**) WT and (**f**) *lm7* leaves. Scale bars = 50 μm. The mean value of (**g**) large veins number, (**h**) small veins number, (**i**) epidermal cell width, (**j**) epidermal cell length, and (**k**) epidermal cell number along leaf-width axis. Significant differences compared with the WT were determined using Student’s *t*-test: ** *p* < 0.01. n.s. means not significant.

To characterize the phenotype of *lm7* in detail, sections of leaves were compared between *lm7* and WT. We observed that the *lm7* had fewer large veins and small veins compared to WT in the mature leaves (Figure 2a–d). The *lm7* had over approximately 25% fewer veins than that of WT (Figure 2g,h). Leaf size is affected by both cell division and expansion. We further studied the relationship between leaf narrowing and leaf epidermal cell number and size. A clear difference in cell size was observed in epidermal cells between WT and *lm7* leaves (Figure 2e,f). The cell width was smaller in the *lm7*; the epidermal cell width in the *lm7* mutant were approximately 15% lower than those in the WT (Figure 2i). The significantly lower cell number was observed in *lm7* compared with that of WT, while no significant difference in cell length was found between WT and *lm7* (Figure 2j,k). Thus, *LM7* leaf size might be modulated by regulating the formation of veins and cell division and expansion.

### 2.3. Cloning of Leaf Width Gene LM7

**Figure 3:**
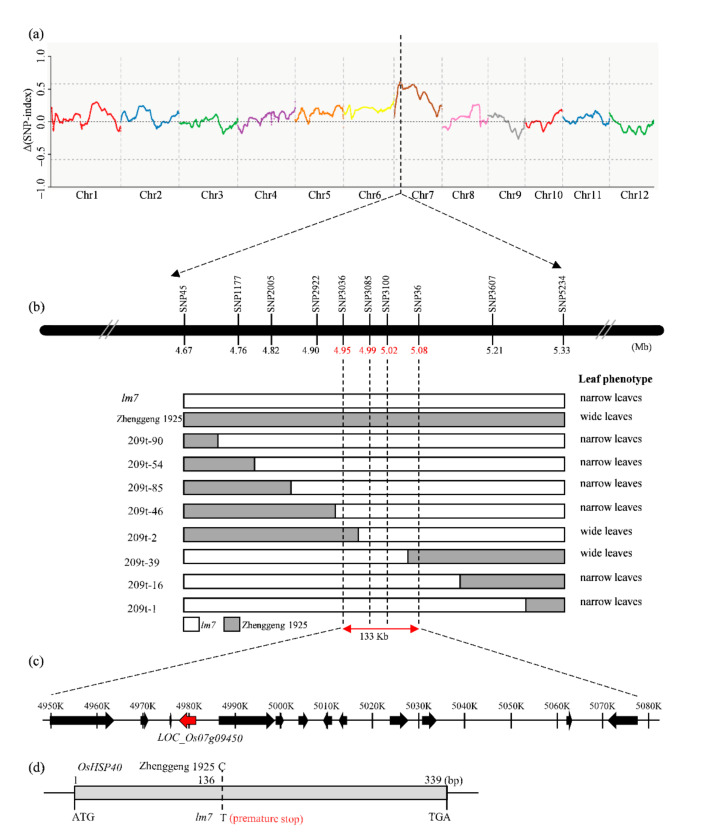
Mapping and cloning of *LM7* by bulked segregant analysis using sequencing. (**a**) Distribution of Δ index of single nucleotide polymorphism (SNP) across 12 chromosomes. Δ (SNP-index) means the absolute value of the difference of SNP index between the bulked pool and Zhenggeng 1925. (**b**) SNP screening of 99 F_2_ individuals originating from *lw7* × Zhenggeng 1925 narrowed down the location of the *LW7* locus to a 133 kb region bounded by markers SNP3036 and SNP36 on chromosome 7. Numbers below the chromosome indicate the physical position of markers. White and gray indicates the *lw7* and Zhenggeng 1925 background respectively. (**c**) Physical position of the *LW7* locus. The arrows represent 13 annotated ORFs in the 133 kb fine-mapping interval according to the rice MSU 7 reference genome. LOC_Os07g09450 in red is the candidate gene for *LW7.* (**d**) CDS structure of candidate gene *LW7/OsHSP40* (LOC_Os07g09450) and mutation site. Sequence analysis revealed a C-to-T nucleotide mutation, which results in forming stop codon in *lw7*.

In order to understand the genetic control of *LM7* for leaf size, we generated a F_2_ population from a cross between *lm7* and wild-type (WT) Zhenggeng 1925 to isolate *LM7* gene. The F_1_ generation plants displayed the normal leaf size (WT type), while the F_2_ generation possessed two kinds of phenotypes: small leaf (*lm7* type) and normal leaf (WT type). The ratio of WT type to *lm7* type phenotype fits into 3:1, suggesting a single recessive gene controlling leaf size. MutMap revealed that several markers with clear linkage on chromosome 7 by whole genome resequencing of phenotypic bulks (normal-bulk and mutant-bulk) from the above mentioned F_2_ population (Figure 3a). The putative mutant locus of *LM7* was located in 133 kb region between SNP3036 and SNP36 using polymorphic markers (Figure 3b). This region contains 13 putative genes according to the rice MSU 7 reference genome system (http://rice.plantbiology.msu.edu) (accessed on 8 January 2020) (Figure 3c; Supplementary Table S1). We determined that one gene LOC_Os07g09450 encoding heat shock protein *OsHSP40* carried sequence variation in coding region between *lm7* and WT plants. A nucleotide substitution from C to T in the coding region of LOC_Os07g09450 was observed between *lm7* and WT, which results in forming stop codon in *lm7* (Figure 3d). There were no base variations in the coding regions of other genes. Therefore, we assumed that the *OsHSP40* is most likely the candidate gene for *LM7*.

### 2.4. OsHSP40 Is the Target Gene for LM7

**Figure 4:**
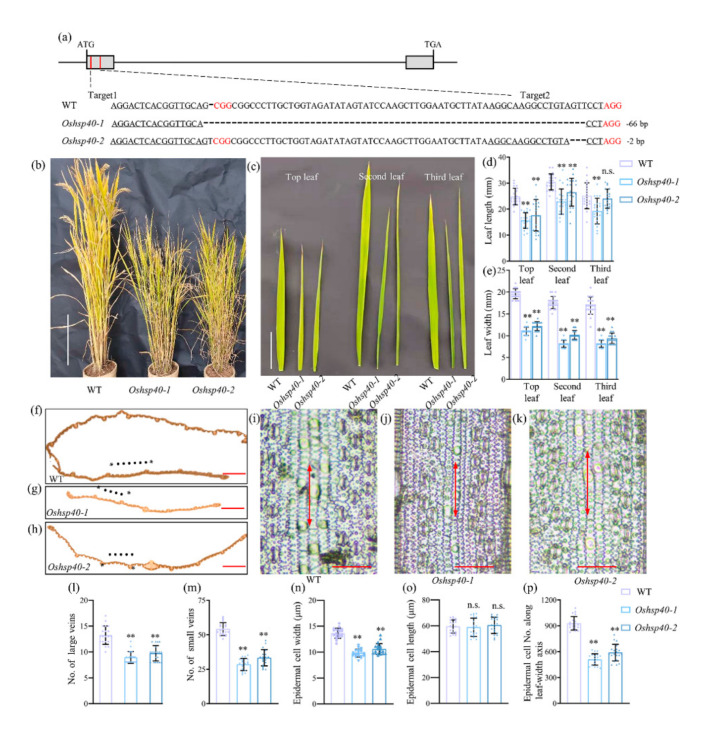
Function verification of the candidate gene *OsHSP40* for *LM7.* (**a**) *Oshsp40-1* and *Oshsp40-2* mutants generated in wild-type (WT) Zhenggeng 1925 by CRISPR/Cas9 approach. A total of 66 and 2 bp nucleotides were deleted in *Oshsp40-1* and *Oshsp40-2* respectively. The two sgRNA:Cas9 target sites are labelled in red lines; gray rectangles indicate exons. (**b**) Representative image of mature plant of WT and *Oshsp40* mutants. Scale bar is 20 cm. (**c**) Representative image of the top, second, and third leaf in WT and *Oshsp40* mutants. Scale bar is 1 cm. The mean value of (**d**) leaf length and (**e**) leaf width. Cross section of (**f**) WT, (**g**) *Oshsp40-1*, and (**h**) *Oshsp40-2* leaves. Asterisks represent large veins and solid circles represent small veins. Scale bar is 1 mm. A clear show of epidermal cells of (**i**) WT, (**j**) *Oshsp40-1*, and (**k**) *Oshsp40-2* leaves. Scale bars = 50 μm. The mean value of (**l**) large veins number, (**m**) small veins number, (**n**) epidermal cell width, (**o**) epidermal cell length, and (**p**) epidermal cell number along leaf-width axis. Significant differences compared with the WT were determined using Student’s *t*-test: ** *p* < 0.01. n.s. means not significant.

To confirm whether the loss of function of *OsHSP40* causes the small leaf phenotype, the *Oshsp40* mutants were generated in wild-type (WT) Zhenggeng 1925 background using CRISPR/Cas9 approach. Two gene knockout lines (*Oshsp40-1* and *Oshsp40-2*) were obtained for the following phenotype evaluation (Figure 4a). The *Oshsp40-1* and *Oshsp40-2* mutants had shorter plant height and smaller leaf size than those of WT (Figure 4b,c). At the heading stage, the plant height of *Oshsp40* mutants was approximately over 20% decreased compared with that of WT. The length of top, second, and third leaf in *Oshsp40* mutants was approximately over 33%, 19%, and 14% decreased compared with that of WT (Figure 4d), respectively, and the leaf width in *Oshsp40* mutants decreased by approximately over 41%, 48%, and 48% (Figure 4e).

To characterize the phenotype of *Oshsp40* mutants in detail, sections of leaf were compared between *Oshsp40* mutants and WT. We observed that the *Oshsp40* mutants had fewer large veins and small veins compared to WT in the mature leaves (Figure 4f–h). The *Oshsp40* mutants had over 25% fewer large veins (Figure 4l) and over 44% fewer small veins than WT (Figure 4m). Leaf size is affected by both cell division and expansion. We further studied the relationship between small leaves and leaf epidermal cell number and size. A clear difference in cell size was observed in epidermal cells between WT and *Oshsp40* mutants (Figure 4i–k). The cell width was smaller in the *Oshsp40* mutants compared with WT. The epidermal cell width in the *Oshsp40* mutants was approximately 22% lower than that in the WT (Figure 4n), and the epidermal cell number along leaf-width axis in the *Oshsp40* mutants was approximately 45% lower (Figure 4p). However, no significant differences in cell length were observed between WT and *Oshsp40* mutants (Figure 4o). Thus, the *OsHSP40* modulated leaf size might be via regulating the formation of the veins and affecting cell division and expansion. 

### 2.5. Characterization of OsHSP40 and Its Expression Pattern

**Figure 5:**
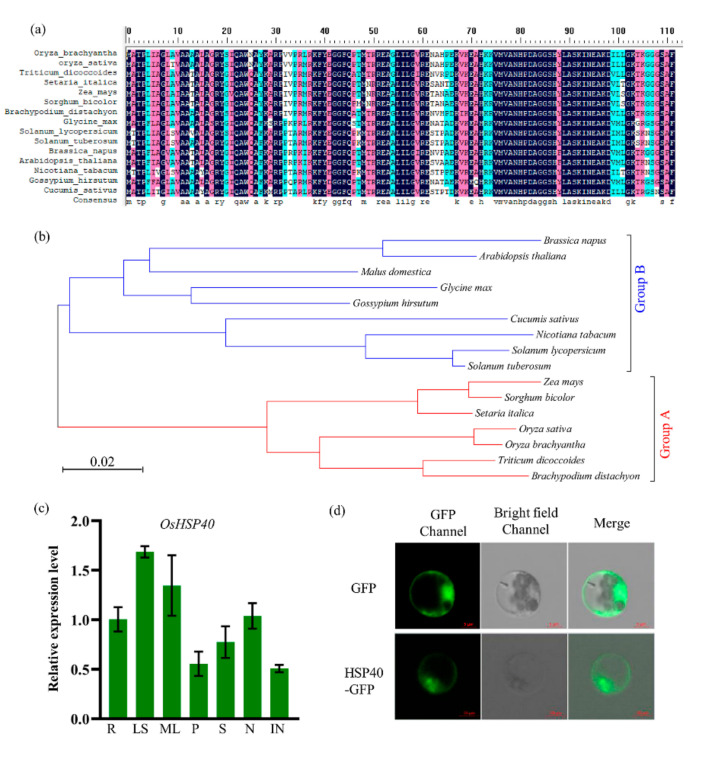
Characterization of *OsHSP40* and its expression pattern. (**a**) Protein sequence alignment of OsHSP40 in different plants. Full-length of amino acid sequences from NCBI were used for analyses. (**b**) Phylogenetic tree showing the relationship between *OsHSP40* homologs in monocots (Group A) and dicots (Group B). (**c**) Relative expression of *OsHSP40* in various rice tissues determined by quantitative RT-PCR in wild type Zhenggeng 1925. R, roots; LS, leaf sheath; ML, mature leaves; P, panicles (10–15 cm); S, seeds (11–20 days after pollination); N, nodes; IN, internodes. Expression is relative to that in the root, the value of which was set as 1. *OsActin* gene was used as the internal control. (**d**) Subcellular localization of *OsHSP40* tagged at the C-terminus with GFP in rice protoplasts.

To reveal the characterization of *OsHSP40* in detail, its phylogenetic analysis and expression pattern were conducted. We observed that heat shock protein *OsHSP40* is a quite conservative and widely distributed proteins in plants. Sequence alignment indicated that OsHSP40 homologs are highly conserved in most parts of the protein (Figure 5a). A phylogenetic tree was constructed using ortholog *OsHSP40* genes from different species, which showed that the genes can be divided into two classes (monocots and dicots) (Figure 5b). A high identity of rice *OsHSP40* with its orthologous genes was observed in other plants, such as *Triticum dicoccoides*, *Brachypodium distachyon*, *Zea mays,* and *Sorghum bicolor*. The expression profile of *OsHSP40* in different tissues of WT Zhenggeng 1925 was conducted using a quantitative RT-PCR (qRT-PCR) approach. We observed that the *OsHSP40* gene displayed constitutive expression in all rice tissues including root, leaf sheath, mature leaf, panicle, seed, node, and internode (Figure 5c); the highest expression was found in the leaf sheath and mature leaves. The analysis of subcellular localization of OsHSP40 showed that OsHSP40 protein is located in cytoplasm (Figure 5d). 

### 2.6. Functional SNP Diversity of OsHSP40

**Figure 6:**
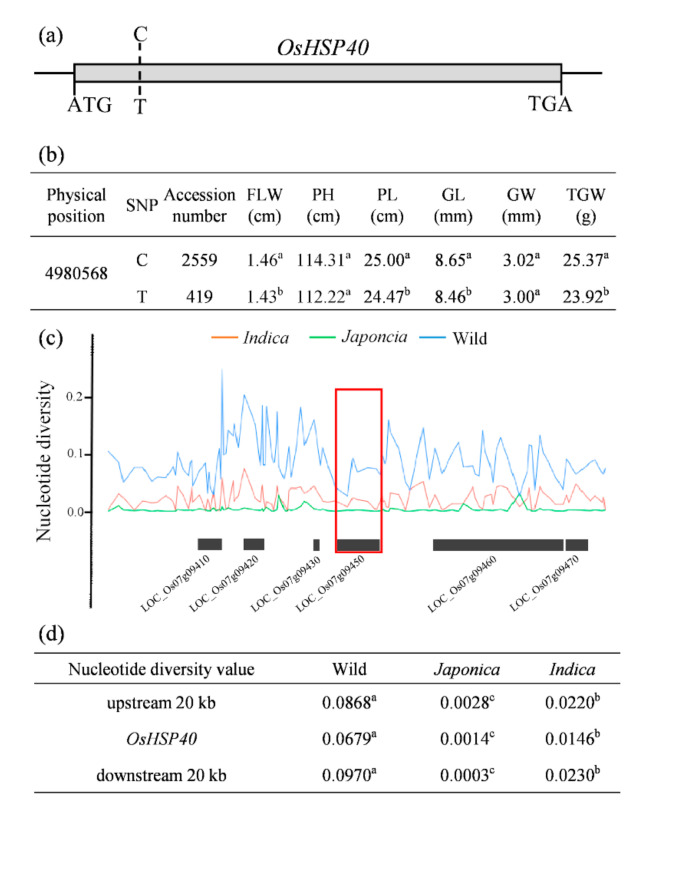
Functional SNP and nucleotide diversity analysis of *OsHSP40.* (**a**) The position of functional SNP in the CDS region of *OsHSP40.* (**b**) Comparisons of phenotype in the accessions with different functional SNPs of *OsHSP40*. FLW, flag leaf width; PH, plant height; PL, panicle length; GL, grain length; GW, grain width; TGW, 1000-grain weight. (**c**) Nucleotide diversity of *OsHSP40* in *japonica*, *indica*, and wild rice. Red box denotes the position of *OsHSP40*. (**d**) Average nucleotide diversity of the 20 kb region surrounding *OsHSP40*. The different letters indicate the significant differences determined using ANOVA test: *p* < 0.05.

To further reveal the natural variation of *OsHSP40*, a total of 2978 rice accessions were further used for haplotype analysis (https://www.rmbreeding.cn/Genotype/haplotype) (accessed on 10 November 2021). One functional SNP (C to T) in the coding region of *OsHSP40* was identified among rice accessions (Figure 6a). We observed that the accessions with C SNP had significantly higher agronomic trait values such as flag leaf width (FLW), plant height (PH), panicle length (PL), grain length (GL), grain weight (GW), and thousand grain weight (TGW) compared with those of accessions with T SNP (Figure 6b; Supplementary Table S2). In order to reveal whether artificial selection of this SNP has contribution to the domestication of *OsHSP40*, we estimated the nucleotide diversity across a 20 kb upstream and downstream genomic region flanking *OsHSP40* in all rice subpopulations, including 1612 cultivated rice accessions and 446 *O. rufipogon* accessions (https://venyao.xyz/ECOGEMS/) (accessed on 10 November 2021) (Figure 6c). The nucleotide diversity value (π) of *OsHSP40* was significantly lower in both *japonica* and *indica* rice compared with that of wild rice (Figure 6d). It suggested that functional *OsHSP40* might be derived from wild rice and low nucleotide diversity in the locus of *OsHSP40* might be the result of artificial selection in *japonica* and *indica* rice.

## 3. Discussion

It has been widely reported that leaf size, such as leaf width and length, is correlated with grain yield [23,24]. Leaf size is a complex trait regulated by a number of factors, and it is an important trait for rice breeding. In this study, one EMS mutant *lm7* related to the short and narrow leaf phenotype was identified in rice. MutMap approach analyses indicated that the *LM7* encodes a heat shock protein *OsHSP40* influencing leaf size. We confirmed that *OsHSP40* was the target gene for *LM7* by mutant analysis. Moreover, the highest expression of *OsHSP40* was found in the leaf sheath and mature leaves in rice, suggesting that it plays a role in leaf growth. Interestingly, the significant differences in plant height, panicle length and grain size were also observed between *Oshsp40* mutants and WT, and functional SNP diversity of *OsHSP40* were observe for grain traits. In this study, the *OsHSP40* regulation on leaf size was mainly conducted, while its regulatory functions on other traits, such as grain size, are deserved investigation in the future. 

Leaf development is a complex process that involves cell division, cell expansion, axis determination, and tissue differentiation and specification [25,26]. In rice, the *nrl1* mutant with narrow leaf width is likely due to its lower number of cells [27]. The narrow leaf phenotype of the *nal9* mutant is due to a significant reduction in the total number of vascular bundles, which causes a reduced cell number in the lateral direction [4]. The *nrl2* mutants showed narrow leaves with a reduction in both the numbers of large and small veins [15]. The *NAL1* mutants exhibited narrow leaf width due to its reduction in cell division and cell expansion with small leaf abaxial epidermal cells and culm parenchyma cells [28,29]. Similarly, the *lm7* and *Oshsp40* mutants had fewer large and small veins, as well as smaller cell width, compared to WT in this study, suggesting that *OsHSP40* modulated leaf size might be via regulating the formation of the veins and cell division and expansion. 

HSP40 is essential for the interaction with HSP70 involved in protein folding, translation, stabilization, and protein translocation across cell membrane [20]. HSP40 recognizes unfolded substrates and deliver them to HSP70, stimulating its ATPase activity, which in turn induces a change in the conformation of the chaperone that stabilizes its interaction with the substrate [30]. Several genes such as *NAL1*, *NAL7*, *NAL21*, *DNL-4,* and *CLSD4* have been reported involving in leaf size in rice [5,8,31,32,33]. Whether *OsHSP40* regulated leaf size in rice by influencing these leaf-related genes needs to be further investigated. Additionally, the HSP70/HSP40 complex is also involved in protein degradation through the ubiquitin-proteasome system [34], aggregated protein clearing through autophagy [35], and mitochondrial DNA and plasmid replication [36,37]. Whether the interactions of OsHSP40 and OsHSP70 are involved in the regulation of leaf size needs further investigation in the future.

In conclusion, in this study, a mutant with smaller leaf phenotype, *lm7*, was obtained using EMS treatment in *japonica* Zhenggeng 1925. We confirmed that the candidate gene *OsHSP40* for *LM7* is involved in the smaller leaf phenotype. After analyzing the leaf structure, we determined that the vein number, cell width, and cell number significantly decreased in the mutants compared with wild type, which caused smaller leaf size. The natural variation of *OsHSP40* contributing to leaf size might be useful for future rice breeding.

## 4. Materials and Methods

### 4.1. Plant Materials and Growth Conditions

The *Leaf Mutant 7* (*lm7)* was isolated from a population of *japonica* inbred line Zhenggeng 1925 and was mutated with a 0.9% ethyl methanesulfonate (EMS) solution. The *japonica* Zhenggeng 1925 was cultivated by Henan Academy of Agricultural Sciences (Zhengzhou, Henan Province, China), and it has excellent grain yield, grain quality, and agronomic traits. The F_2_ population, developed from a cross between *lm7* (female parent) and wild-type (WT) Zhenggeng 1925 (male parent), was used for bulk segregant analysis (BSA). Two mutants *Oshsp40-1* and *Oshsp40-2* were generated using the CRISPR/Cas9 system in the *japonica* Zhenggeng 1925 background. All plants were grown in the experimental fields of Henan Academy of Agricultural Sciences. Field management was performed in accordance with the local standard methods. Seed sowing was conducted on May 5 and then seedlings were transplanted on June 14 in fields with a plant row spacing of 14 × 17 cm. Fertilizer at the level of 225 kg/hm^2^ was applied with the ratio of nitrogen:phosphorus:potassium being 2:1:1. Water management was conducted according to the water demand of rice.

### 4.2. CRISPR/Cas9 Vector Construction and Plant Transformation

The CRISPR/Cas9 binary vector pHUE411 carrying two gRNAs targeting (5′-AGGACTCACGGTTGCAGCGG-3′; 5′-AGGCAAGGCCTGTAGTTCCT-3′) was transformed into *Agrobacterium* strain EHA105, and *Agrobacterium*-mediated method was used to transform immature embryos of Zhenggeng 1925. The genomic DNA was extracted from transgenic seedlings, and the primers for cloning fragments with targets were listed in Table S3. At last, the PCR products were sequenced and blasted to identify homozygous mutants.

### 4.3. Characterization of Mutant Phenotype

At the heading stage, the plants of wild type (WT) Zhenggeng 1925, and *lm7* and *Oshsp40* mutants, were selected to investigate their plant height, leaf width, panicle length, grain size, and 1000-grain weight. Meanwhile, their mature leaf blades were used for microscopy observation. To investigate the morphology of the vascular bundles of the leaf blade, tissues were decolorized in a graded ethanol series and observed using stereoscope (ZEISS Stemi 508, Jena, Germany) [5]. To investigate the width of leaf epidermal cells, tissues were soaked in solution (30% H_2_O_2_: glacial acetic acid = 1:1) and then torn off the leaf epidermis with sharp tweezers. Next, the leaf epidermis was stained with 1% toluidine blue for 1–2 min and observed using light microscope (NEXCOPE NE610, Shenzhen, China) [32].

### 4.4. Bulk Segregant Analysis

For mapping of the *LM7* locus, 72 mutant-type plants and 110 normal-type plants were selected from the abovementioned F_2_ population from a cross between *lm7* and Zhenggeng 1925 to constitute the mutant-bulk and normal-bulk, respectively. DNA were extracted from young leaf tissues using the cetyltrimethylammonium bromide (CTAB) method [38]. Both mutant-bulk and normal-bulk along with parents *lm7* and Zhenggeng 1925 were sequencing by GENOSEQ Co., Ltd., Wuhan, China. Candidate genes were predicted using the positions from the Rice Genome Annotation Project MSU7 database (Rice Genome Browser: http://rice.plantbiology.msu.edu) (accessed on 8 January 2020).

### 4.5. Sequence Alignment of the OsHSP40 Protein and Phylogenetic Analysis

Protein sequences were retrieved from the NCBI database using blastp program (https://blast.ncbi.nlm.nih.gov/Blast.cgi) (accessed on 10 November 2021) and aligned using DNAMAN software. Phylogenetic tree was constructed using the MEGA7 software by employing the neighbor-joining method with 1000 bootstrap replicates.

### 4.6. Subcellular Localization of OsHSP40

For observation of GFP-*OsHSP40* in rice protoplasts, the CaMV 35S promoter-driven GFP-*OsHSP40* was cloned into the pCambia1305-GFP vector according to the manufacturer’s instructions (Vazyme, Nanjing, China). Fluorescence images were captured with a confocal laser scanning microscope (LSM 780; Carl Zeiss, Berlin, Germany).

### 4.7. Quantitative Reverse Transcription PCR Analysis

Total RNA was extracted from the various tissues using the TransZol Plant Kit (Transgen, Beijing, China) according to the manufacturer’s protocol. Quantitative RT-PCR was carried out according to the method described by He et al. [39]. The primers used for quantitative RT-PCR are listed in Supplementary Table S3. Normalized transcript levels of gene expression were calculated using the comparative *C_T_* method [40]. Three biological replicates were performed.

### 4.8. Nucleotide Diversity Analysis

The nucleotide diversity of *OsHSP40* and its flanking regions were obtained from the ECOGEMS database [41] and RFGB database [42].

### 4.9. Data Analysis

Experimental data were analyzed using the EXCEL2019 software, and significant differences among samples were compared using Student’s *t*-test or ANOVA test at the 5% and 1% levels of probability.

## Data Availability

Not applicable.

## Supplementary Materials

The following supporting information can be downloaded at: https://www.mdpi.com/article/10.3390/ijms23084446/s1.
