# Supplementary material for: Leaf Mutant 7 Encoding Heat Shock Protein OsHSP40 Regulates Leaf Size in Rice
Source: Int J Mol Sci. 2022 Apr 18;23(8):4446. doi: 10.3390/ijms23084446 (PMC9027358; doi:10.3390/ijms23084446)
Supplement: Supplementary file 1 [file ijms-23-04446-s001.zip › Supplementary Tables S1 and S3.pdf]

Table S1. Candidate genes predicted for LM7.

Candidate Gene ID	Gene Function Annotation
LOC_Os07g09400	RNA ligase isoform 1
LOC_Os07g09420	ATPase
LOC_Os07g09430	expressed protein
LOC_Os07g09450	heat shock protein DnaJ
LOC_Os07g09460	expressed protein
LOC_Os07g09470	ATPase
LOC_Os07g09480	ATPase
LOC_Os07g09490	ATPase
LOC_Os07g09500	expressed protein
LOC_Os07g09520	expressed protein
LOC_Os07g09530	ankyrin repeat family protein
LOC_Os07g09560	ankyrin repeat family protein
LOC_Os07g09580	aminoacyl-tRNA synthetase

Table S3. The primer pairs used in this study.

Primer Name	Primer Sequence (5′–3′)	Purpose
HSP-F	ATGGCTACACCACTCATAGCAGG	Gene cloing
HSP-R	TCAAAAGGCTGACCCCCCT	
HSPcp-F	CAGCGGGTTGTCCGAAACT	Verification for CRISPR-knockout plants
HSPcp-R	GGGCAGGGATCAGGAAAAGT	
HSPqRT-F	AGTACCGGAAATGGCTACACCAC	qRT-PCR
HSPqRT-R	TGCGCATCCTAGGAACTACAGG	
NAL21qRT-F	ATTCTAAAGGCCCCGAAATTTG	
NAL21qRT-R	TCACGATCCACAACTAGATAGC	
NAL7qRT-F	CAAGAACATCACCGGCAAGA	
NAL7qRT-R	CGATTTGATCAAGGACCATGCT	
CLSD4qRT-F	TCAGTAGTGTAGTGGTGTCGAGTTCA	
CLSD4qRT-R	GCACTCCTTCATGTGAGCTTCA	
NAL1qRT-F	TGCAGTGTCCGCTCAATAGC	
NAL1qRT-R	GACCAGAGCTTCTGCCAACTTT	
DNL4qRT-F	TTGGAGCTTGTGGAGATGACAGC	
DNL4qRT-R	TCGACCAAGCATGCACACTGAG	
OsActin-F	AGGAAGGCTGGAAGAGGACC	
OsActin-R	CGGGAAATTGTGAGGGACAT	
